# A Wireless Wearable Doppler Ultrasound Detects Changing Stroke Volume: Proof-of-Principle Comparison with Trans-Esophageal Echocardiography during Coronary Bypass Surgery

**DOI:** 10.3390/bioengineering8120203

**Published:** 2021-12-08

**Authors:** Jon-Émile Stuart Kenny, Geoffrey Clarke, Matt Myers, Mai Elfarnawany, Andrew M. Eibl, Joseph K. Eibl, Bhanu Nalla, Rony Atoui

**Affiliations:** 1 Health Sciences North Research Institute, Sudbury, ON P3E 2H2, Canada; gclarke@flosonicsmedical.com (G.C.); aeibl@flosonicsmedical.com (A.M.E.); jeibl@flosonicsmedical.com (J.K.E.); bnalla@nosm.ca (B.N.); rony.atoui@gmail.com (R.A.); 2 Flosonics Medical, 325 Front Street, Toronto, ON M5V 2Y1, Canada; mmyers@flosonicsmedical.com (M.M.); melfarnawany@flosonicsmedical.com (M.E.); 3 Northern Ontario School of Medicine, Sudbury, ON P3E 2C6, Canada

**Keywords:** fluid responsiveness, carotid doppler, functional hemodynamic monitoring, trans-esophageal echocardiography

## Abstract

Background: A novel, wireless, ultrasound biosensor that adheres to the neck and measures real-time Doppler of the carotid artery may be a useful functional hemodynamic monitor. A unique experimental set-up during elective coronary artery bypass surgery is described as a means to compare the wearable Doppler to trans-esophageal echocardiography (TEE). Methods: A total of two representative patients were studied at baseline and during Trendelenburg position. Carotid Doppler spectra from the wearable ultrasound and TEE were synchronously captured. Areas under the receiver operator curve (AUROC) were performed to assess the accuracy of changing common carotid artery velocity time integral (ccVTI_∆_) at detecting a clinically significant change in stroke volume (SV_∆_). Results: Synchronously measuring and comparing Doppler spectra from the wearable ultrasound and TEE is feasible during Trendelenburg positioning. In two representative cardiac surgical patients, the ccVTI_∆_ accurately detected a clinically significant SV_∆_ with AUROCs of 0.89, 0.91, and 0.95 when single-beat, 3-consecutive beat and 10-consecutive beat averages were assessed, respectively. Conclusion: In this proof-of-principle research communication, a wearable Doppler ultrasound system is successfully compared to TEE. Preliminary data suggests that the diagnostic accuracy of carotid Doppler ultrasonography at detecting clinically significant SV_∆_ is enhanced by averaging more cardiac cycles.

## 1. Introduction

We have previously described a wireless Doppler ultrasound patch that adheres to the neck over the common carotid artery, maintains a constant angle of insonation, and accurately measures Doppler metrics such as the velocity time integral (VTI) and corrected carotid flow time (ccFT) [1]. One clinical application of this unique biosensor, particularly within the sphere of acute care medicine, is functional hemodynamic monitoring (FHM) [2]. FHM is predicated upon accurately measuring change in—rather than absolute values of—stroke volume (SV) [3,4]. For example, should the provision of intravenous fluid be followed by minimal change in SV (SV_∆_), the treating clinician intimates a heart that is resistant to additional intravenous fluids [5]; he or she then avoids over-prescribing crystalloid therapy that is arguably futile and/or harmful [6,7].

Yet measuring SV_∆_ at the bedside is a challenging aspect of FHM. Quantitative Doppler ultrasonography of the descending aorta and left ventricular outflow tract (LVOT) are used [8,9], but these processes are time-intensive and require advanced training [1]. Therefore, peripheral arteries have been exploited as surrogates of SV_∆_ [10] as peripheral arteries, like the common carotid [11,12], are typically easier to insonate. Nevertheless, key drawbacks to hand-held Doppler measurements are human error [13,14] and relatively little guidance on the number of cardiac cycles to measure [15].

As solutions to the aforementioned challenges, we hypothesized that the novel, wearable Doppler biosensor could be employed in patients undergoing coronary bypass surgery. Further, as a clinical proof-of-principle, we also hypothesized that the common carotid artery velocity time integral (ccVTI), measured by the hands-free, wireless Doppler device, would accurately detect +10% SV_∆_ as measured by synchronously-obtained trans-esophageal echocardiography (TEE). Lastly, we hypothesized that the ability of the carotid artery to detect significant SV_∆_ would improve as more cardiac cycles are averaged.

## 2. Materials and Methods

### 2.1. Patients

We report representative data from 2 patients that were undergoing elective coronary artery bypass surgery. Written and informed consent was obtained and the study was approved by the local institutional review board (IRB), that is, the Research Ethics Board of Health Sciences North. We analyzed one patient with an increase and one with no significant change in LVOT VTI during Trendelenburg positioning. In addition, one patient had atrial fibrillation during the maneuver.

In both of the patients, prior to ultrasound recording, radial artery, peripheral, and internal jugular venous catheters were inserted under local anesthesia. Additionally, electrocardiogram and pulse oximetry were recorded. Anesthesia was induced with 0.5 mg/kg propofol, 1.2 mg/kg rocuronium, and 1.0 μg/kg sufentanil and maintained with sevoflurane of 0.5 to 0.7 minimum alveolar concentration. Continuous IV infusions of both 3 mL/kg/h saline and 0.2 μg/kg/h sufentanil were maintained throughout the study. 

The lungs were ventilated in a volume-controlled mode of ventilation with the following baseline settings: tidal volume (VT) 8 mL/kg of lean body weight, respiratory rate 15 breaths per minute, PEEP 5 cm H_2_O, I:E ratio 1:2 without inspiratory pause, and FiO_2_ 0.5. 

### 2.2. Ultrasound & Stroke Volume Monitoring

**Figure 1:**
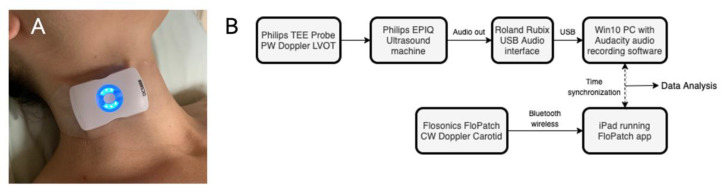
(**A**). The wireless, wearable Doppler ultrasound on a volunteer. (**B**). The experimental set-up with the novel, wearable biosensor (FloPatch^™^). PW is pulse wave; LVOT is left ventricular outflow tract; CW is continuous wave; app is application; USB is universal serial bus; Win10PC is Windows 10 personal computer.

The ultrasound patch (Flosonics Medical, Sudbury, ON, Canada) is a wearable, wireless, FDA-cleared, continuous-wave 4 MHz ultrasound (see Figure 1A). Adhesive straps fix the transducer angle relative to carotid blood flow [16]. The LVOT VTI measurements were made by a cardiac anesthesiologist using a Phillips Epiq (Cambridge, MA, USA) 2.9 MHz trans-esophageal probe in the trans-gastric window. The insonation angle was 0 degrees and sample window 4 mm. The external Doppler audio output from the cart-based imaging system was fed into an audio recorder (Roland Corporation, Los Angeles, CA, USA) and visualised using an open access audio-recording program (Audacity, © 2021-2021 Audacity Team). The Doppler audio output from the trans-esophageal echocardiogram was fed into the Roland audio recorder and recorded for offline analysis, which included exact synchronization with the carotid artery Doppler spectra (see Figure 1B). 

From both the carotid and left ventricular outflow tract, the simultaneous VTI of each cardiac cycle was calculated. The VTI for a single cardiac cycle represents the distance that is traveled by the blood, in centimeters per beat. Regarding the LVOT and assuming constant aortic valve diameter, the variation in VTI is directly proportional to—and interchangeable with—changing stroke volume, SV_∆_ [9].

### 2.3. Preload Modification

Prior to skin incision, 30 s baseline Doppler measurements were obtained with the patient in the supine position. The head of the bed was then lowered to 15 degrees below the horizon, as previously described [17], for an additional 90 s. During both the baseline and head-down positioning, the Doppler spectrogram for each cardiac cycle was measured synchronously between the left ventricular outflow tract that was obtained via TEE and common carotid artery recorded from the wearable Doppler ultrasound. 

### 2.4. Statistical Analysis

The Doppler spectra of synchronous cardiac cycles captured at the common carotid artery and left ventricular outflow tract were compared at 3 levels of analysis: (1) beat-to-beat, (2) averages of 3 consecutive cardiac cycles, and (3) averages of 10 consecutive cardiac cycles. More specifically, for a given patient, each heartbeat in the baseline region of the assessment was compared with each beat in the Trendelenburg region. For each given pair of heart beats, the percent change of carotid VTI was evaluated for its ability to detect a +10% change in LVOT VTI (i.e., SV_∆_). This analysis was repeated using 3-beat means and 10-beat means and the area under the receiver operator curve (AUROC) for detecting +10% SV_∆_ using the synchronously-captured change in common carotid VTI (ccVTI_∆_) was calculated at each level of analysis. As well, the optimal ccVTI_∆_ threshold for detecting +10% SV_∆_ was obtained using the Youden Index.

## 3. Results

**Figure 2:**
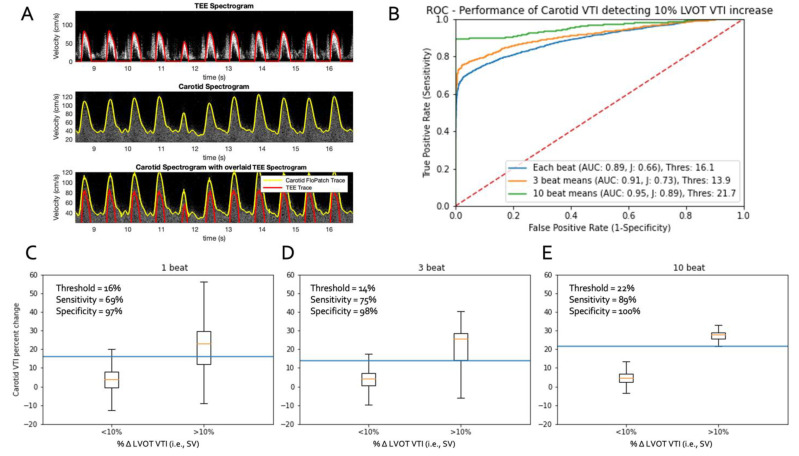
Results from the proof-of-principle study. (**A**) Simultaneously acquired Doppler spectra from the wearable Doppler and the trans-esophageal echocardiogram (TEE) during arrhythmia from one of the studied patients. (**B**) shows the area under the receiver operator curves for beat-to-beat, 3 consecutively averaged and 10 consecutively averaged cardiac cycles for the two patients. (**C**–**E**) shows the boxplots and optimal ccVTI_∆_ thresholds for discriminating +10% SV_∆_ from <10% SV_∆_ across the different degrees of consecutive beat averaging.

A total of two representative patients were selected for proof-of-principle analysis, one of whom had dysrhythmia. The first was a 79-year-old man and the second an 81-year-old man with known atrial fibrillation. Each underwent successful, elective, double and triple coronary artery bypass, respectively, without complication. Representative, synchronously measured Doppler spectra from one of the patients are shown in in Figure 2A.

A total of 4695 single-beat baseline-to-single-beat Trendelenburg combinations were generated between the two patients. A ccVTI_∆_ ≥ 16% correctly identified 1535 of the 2224 ≥ 10% SV_∆_ combinations giving a 69% sensitivity while a ccVTI_∆_ < 16% correctly excluded 2402 of the 2498 < 10% SV_∆_ combinations yielding a 97% specificity. For the single-beat level of analysis, the area under the receiver operator curve was 0.89 (Figure 2B,C). 

A total of 4287 three-beat baseline-to-three-beat Trendelenburg combinations were generated between the two patients. A ccVTI_∆_ ≥ 14% correctly identified 1409 of the 1874 ≥ 10% SV_∆_ combinations producing a 75% sensitivity while ccVTI_∆_ < 14% correctly excluded 2354 of the 2413 < 10% SV_∆_ combinations giving a 98% specificity. For the three-beat level of analysis, the area under the receiver operator curve was 0.91 (Figure 2B,D). 

A total of 2985 10-beat baseline-to-10-beat Trendelenburg combinations were generated between the two patients. A ccVTI_∆_ ≥ 22% correctly identified 783 of the 880 ≥ 10% SV_∆_ combinations for an 89% sensitivity, while a ccVTI_∆_ < 22% correctly excluded all 2105 < 10% SV_∆_ combinations for a specificity of 100%. At the 10-beat analysis level, the area under the receiver operator curve was 0.95 (Figure 2B,E). 

## 4. Discussion

In this proof-of-principle research communication, we have demonstrated a novel experimental set-up whereby carotid Doppler spectra, captured by a unique, wearable biosensor, are synchronously compared to the left ventricular outflow tract Doppler spectra measured via trans-esophageal echocardiography. Further, clinically significant SV_∆_ induced by the Trendelenburg maneuver was accurately detected in the common carotid artery via the wearable Doppler and its accuracy improved as more consecutive cardiac cycles were averaged. 

Ostensibly, the wearable Doppler ultrasound mitigates the clinical challenges that are described at the outset of this brief communication. First, the wearable sensor is adherent and, therefore, largely avoids human measurement inaccuracies. For example, a 5° manual insonation error bestows a 15% velocity error at 60° [13]. In addition, the blunted parabolic profile in the common carotid artery causes velocity gradient broadening [14] which may increase sampling error across the vessel lumen, especially when measurements are repeated. In a human model of hemorrhage and resuscitation comprising approximately 50,000 cardiac cycles, we found a strong linear correlation between SV_∆_ that was measured by non-invasive pulse contour analysis and ccVTI_∆_ that was assessed by the wearable Doppler ultrasound [18]. Yet, a recent investigation found only a moderate relationship between carotid artery blood flow that was quantified by hand-held Doppler and SV_∆_ that was assessed by trans-pulmonary thermodilution in cardiac surgery patients [19]. The authors postulated that autoregulation and/or human factors may have diminished the relationship between the SV and hand-held ultrasonographic carotid artery blood flow assessment. While our proof-of-principle research communication cannot definitively solve this clinical discrepancy, patient enrollment is on-going. Should the novel biosensor described herein show stronger concordance between SV_∆_ and ccVTI_∆_, it suggests that human measurement variation plays a significant role in carotid artery blood flow assessment accuracy [13,14,20,21] in cardiac surgery patients.

Second, we believe that the described experimental paradigm is promising because it illustrates that averaging more beats in the carotid artery improves its relationship with the LVOT VTI. We have previously shown that the inherent physiological variation in the ccVTI that is induced by the respiratory cycle increases the sample size of cardiac cycles required to detect change with statistical confidence [15]. Thus, analyzing the relationship between the carotid artery and left ventricle with varying degrees of granularity may explain discordant clinical data [22,23]. 

The primary clinical limitation of this study is its small sample size. Nevertheless, we report this brief communication as proof-of-principle of an experimental set-up with a novel biosensor. Thus, no definitive clinical conclusions can be drawn from this report. Further, we have reported only the accuracy of the ccVTI for detecting a significant SV_∆_. Neglecting the diameter of the carotid artery precludes measurement of the total flow. We omit carotid diameter measurement because very small errors are amplified to the second power; for example, in a 6 mm (mm) carotid artery, a 1 mm measurement error creates a 30% flow error [24]. Nevertheless, measuring artery diameter may increase the sensitivity of flow assessment, particularly in hypotensive patients [25]. Lastly, this paradigm is limited to functional hemodynamic monitoring which is restricted to relatively rapid pre—post measurements—on the order of 60–120 s [26,27]. Comparisons between SV and carotid flow over much longer time windows are more likely to diverge as a function of changing body-to-carotid artery impedances.

## 5. Conclusions

In conclusion, a novel, wireless, ultrasound patch coupled with a unique experimental paradigm in coronary artery bypass surgery patients might elucidate the relationship between acutely changing ccVTI and SV. Concordance between the two before and during an acute preload change suggests that both minimizing human measurement variability and increasing the number of averaged cardiac cycles might be two critical variables when using the common carotid artery as a transient window to the left ventricle. 

## Data Availability

The datasets used and/or analysed during the current study are available from the corresponding author on reasonable request.
